# Chikungunya virus vaccine: a decade of progress solving epidemiological dilemma, emerging concepts, and immunological interventions

**DOI:** 10.3389/fmicb.2024.1413250

**Published:** 2024-07-22

**Authors:** Mohd Sayeed Shaikh, Md. Faiyazuddin, Mubasshera Sabir Khan, Shahbaz K. Pathan, Imran J. Syed, Amol D. Gholap, Mohammad Shabib Akhtar, Ranjit Sah, Rachana Mehta, Sanjit Sah, D. Katterine Bonilla-Aldana, Camila Luna, Alfonso J. Rodriguez-Morales

**Affiliations:** ^1^Y. B. Chavan College of Pharmacy, Aurangabad, Maharashtra, India; ^2^School of Pharmacy, Al – Karim University, Katihar, India; ^3^Centre for Global Health Research, Saveetha Institute of Medical and Technical Sciences, Chennai, Tamil Nadu, India; ^4^Medmecs Medical Coding & Billing Services, Universal Business Park, Mumbai, Maharashtra, India; ^5^SBSPM’s B. Pharmacy College, Beed, Maharashtra, India; ^6^Department of Pharmaceutics, St. John Institute of Pharmacy and Research, Palghar, Maharashtra, India; ^7^Department of Clinical Pharmacy, College of Pharmacy, Najran University, Najran, Saudi Arabia; ^8^Green City Hospital, Kathmandu, Nepal; ^9^Research Unit, Department of Microbiology, Dr. DY Patil Medical College, Hospital and Research Centre, DY Patil Vidyapeeth, Pune, Maharashtra, India; ^10^Department of Public Health Dentistry, Dr. D. Y. Patil Dental College and Hospital, Dr. D. Y. Patil Vidyapeeth, Pune, Maharashtra, India; ^11^Dr Lal PathLabs Nepal, Kathmandu, Nepal; ^12^Medical Laboratories Techniques Department, AL-Mustaqbal University, Hillah, Babil, Iraq; ^13^Clinical Microbiology, School of Dental Science, Manav Rachna International Institute of Research and Studies, Faridabad, Haryana, India; ^14^SR Sanjeevani Hospital, Siraha, Nepal; ^15^College of Medicine, Korea University, Seoul, Republic of Korea; ^16^Faculty of Health Sciences, Universidad Científica del Sur, Lima, Peru; ^17^Grupo de Investigación Biomedicina, Faculty of Medicine, Fundación Universitaria Autónoma de las Américas-Institución Universitaria Visión de las Américas, Pereira, Colombia; ^18^Gilbert and Rose-Marie Chagoury School of Medicine, Lebanese American University, Beirut, Lebanon

**Keywords:** chikungunya, prevention, vaccines, immunology, epidemiology, RNA vaccines

## Abstract

Chikungunya virus (CHIKV), a single-stranded RNA virus transmitted by Aedes mosquitoes, poses a significant global health threat, with severe complications observed in vulnerable populations. The only licensed vaccine, IXCHIQ, approved by the US FDA, is insufficient to address the growing disease burden, particularly in endemic regions lacking herd immunity. Monoclonal antibodies (mAbs), explicitly targeting structural proteins E1/E2, demonstrate promise in passive transfer studies, with mouse and human-derived mAbs showing protective efficacy. This article explores various vaccine candidates, including live attenuated, killed, nucleic acid-based (DNA/RNA), virus-like particle, chimeric, subunit, and adenovirus vectored vaccines. RNA vaccines have emerged as promising candidates due to their rapid response capabilities and enhanced safety profile. This review underscores the importance of the E1 and E2 proteins as immunogens, emphasizing their antigenic potential. Several vaccine candidates, such as CHIKV/IRES, measles vector (MV-CHIK), synthetic DNA-encoded antibodies, and mRNA-lipid nanoparticle vaccines, demonstrate encouraging preclinical and clinical results. In addition to identifying potential molecular targets for antiviral therapy, the study looks into the roles played by Toll-like receptors, RIG-I, and NOD-like receptors in the immune response to CHIKV. It also offers insights into novel tactics and promising vaccine candidates. This article discusses potential antiviral targets, the significance of E1 and E2 proteins, monoclonal antibodies, and RNA vaccines as prospective Chikungunya virus vaccine candidates.

## 1 Introduction

Chikungunya virus (CHIKV), a compact enveloped virus measuring approximately 60–70 nm, is a single-stranded RNA virus that spreads through *Aedes* mosquitoes (Erasmus et al., 2016). Moreover, CHIKV infection can also cause neuropathology, especially in infants and elderly individuals, which can be fatal (Priya et al., 2014). Currently, real-time PCR identification of viral RNA and serological analysis for the presence of IgM and IgG antibodies form the basis of laboratory diagnosis of CHIKV infection (Chandley et al., 2023). Treatment of CHIKV infection involves the use of anti-inflammatory drugs and analgesics, which are inadequate (Javaid et al., 2022). Despite its increased global and national disease burden, IXCHIQ is the only licensed vaccine and effective antiviral therapy approved by the US FDA to treat or prevent CHIKV infection (Lucey, 2023). The lack of herd immunity in developing countries, which are endemic to CHIKV infection, presents an imminent risk for the spread of large-scale outbreaks. Therefore, it is essential to understand the development of anti-CHIKV immunity through vaccination or passive immunization techniques (Schrauf et al., 2020). CHIKV is an enveloped alphavirus that enters host cells via receptor-mediated internalization. The RNA genome of CHIKV encodes four nonstructural proteins (nsP1 to nsP4) that are required for virus replication and three structural proteins (capsid, E1 and E2) together with two small cleavage products (E3 and 6 K). The E1 and E2 glycoproteins regulate the entry of viruses into host cells; E1 is involved in the fusion of the virus with the cell membrane, whereas E2 interacts with receptors on cells and aids in cell attachment (Javaid et al., 2022; Henderson Sousa et al., 2023; Varikkodan et al., 2023). The E2 glycoprotein has been identified as the primary target for the anti-CHIKV antibody response throughout the illness (Chandley et al., 2023). E1 plays an important role in membrane fusion, and E2 is responsible for receptor binding. Therefore, the E1 and E2 proteins are often selected as immunogens in vaccines. This indicates that CHIKV E2-E1 has attractive antigenic potential (Cho et al., 2008; Weber et al., 2017; da Cruz Silva et al., 2022). David B. Weiner et al. formulated a synthetic DNA vaccine to create targeted immunity against CHIKV expressing its envelope glycoprotein. It successfully induced robust immune responses in mice and Rhesus Macaques (Muthumani et al., 2008). Yu Wei et al. developed an mRNA-lipid nanoparticle (mRNA-LNP) vaccine expressing the CHIKV E2-E1 antigen (Ge et al., 2022).

**Figure 1:**
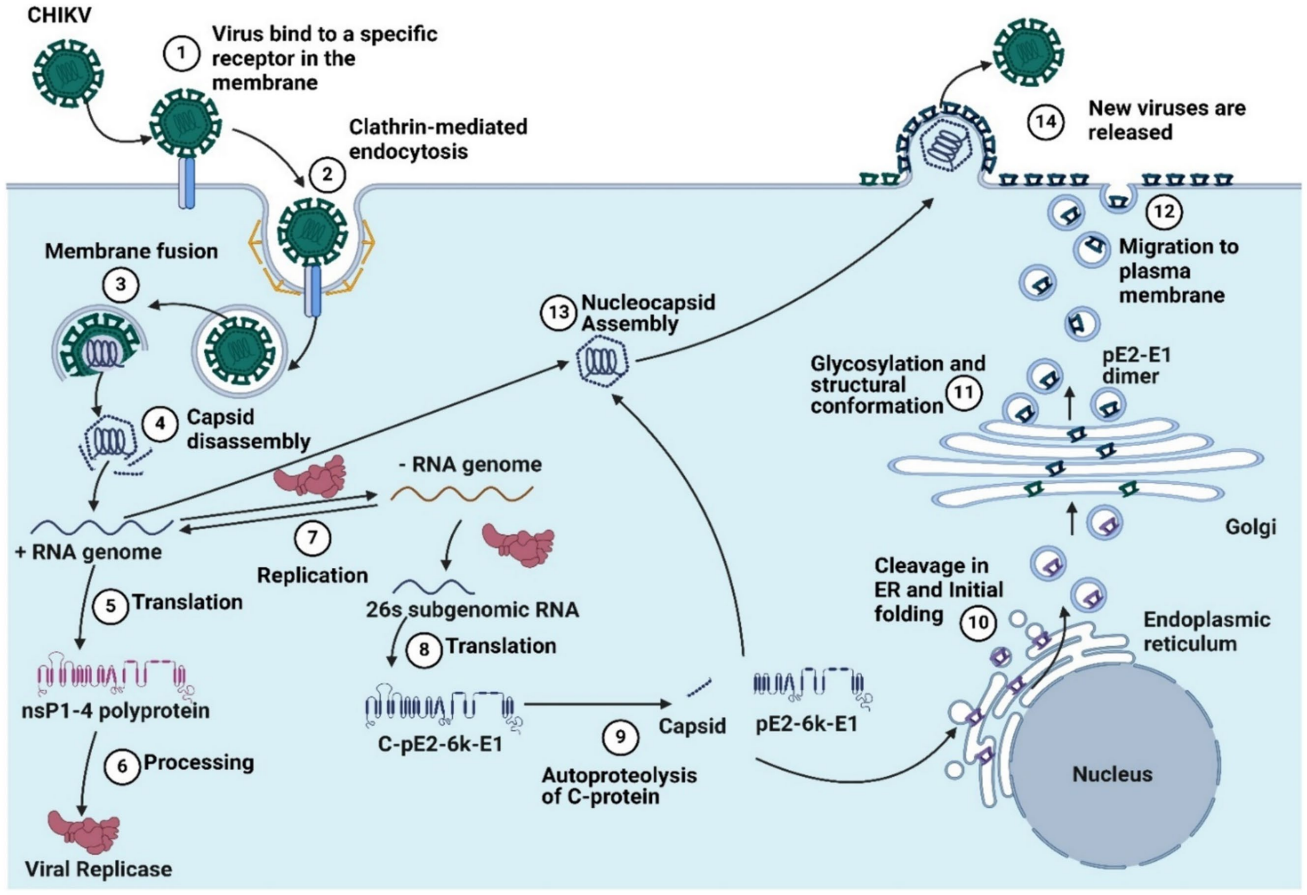
The chikungunya virus replication cycle includes (1) virus binding to a specific receptor in the membrane, (2) clathrin-mediated endocytosis, (3) membrane fusion, (4) capsid disassembly, (5) translation, (6) processing, (7) replication, (8) 26’s subgenomic RNA translation, (9) autoproteolysis, (10) cleavage in the endoplastic reticulum and initial folding, and (11). Glycosylation and structural conformation, (12) migration to the plasma membrane, (13) nucleocapsid assembly, and (14) the release of new viruses (Created by using Biorender.com).

Using this knowledge, a reverse vaccinology approach can be successfully employed to design a safe and effective anti-CHIKV vaccine (Masrinoul et al., 2014; Segato-Vendrameto et al., 2023). Besides the development of monoclonal antibodies, vaccination is another approach for controlling CHIKV infection. Various vaccine candidates are currently being explored for mediating immunity to CHIKV, such as using different types like live attenuated virus, killed vaccines, nucleic-acid-based (DNA/RMA) vaccines, virus-like particle vaccines, chimeric vaccine, subunit vaccines and adenovirus vectored vaccines (Ge et al., 2022; Schmidt et al., 2022a). Live attenuated vaccines effectively trigger immune responses but carry the risk of virus reversion (Abeyratne et al., 2019). Virus-like particle vaccines are safer but mainly elicit humoral responses and are costly. RNA-based vaccines, which have proven effective during the COVID-19 pandemic, offer a rapid response to emerging infections (Schmidt et al., 2022a; Tsai et al., 2023). RNA vaccines can induce robust immune responses with improved safety, making them promising candidates for CHIKV vaccines (Shaw et al., 2019). RNA vaccine candidates include nonreplicating RNAs (nrRNAs) with synthetic modifications, self-amplifying RNAs (saRNAs) and trans-amplifying RNAs (taRNAs), which are derived from positive-sense viruses (Schmidt et al., 2022a). The chikungunya virus replication cycle is presented in Figure 1. Charalambos D. Partidos et al. have developed a live attenuated CHIKV vaccine (CHIKV/IRES) that is highly attenuated yet immunogenic in mouse models and incapable of replicating in mosquito cells (Chu et al., 2013). Ramsauer et al. (2019) developed a CHIKV vaccine using a measles vector (MV-CHIK) that effectively triggered immune responses, even in individuals with existing measles immunity. Clinical trial results have shown promising outcomes in terms of safety, tolerability, and immunogenicity. Their research revealed that this vaccine not only triggered a more potent neutralizing antibody response but also elicited stronger cellular immune responses, particularly CD8+ T-cell responses, than recombinant protein antigens, showing its enhanced efficacy over recombinant protein CHIKV vaccine candidates (Akahata and Nabel, 2012; Ge et al., 2022).

Humoral immunity plays an important role in virus clearance. Viremia can be rapidly cleared in wild-type mice infected with an attenuated CHIKV strain but not in B-cell deficient (μMT) mice. Priya et al. (2013) reported that Toll-like receptors (TLRs) are crucial for the impact of CHIKV on neuronal cells. TLRs are vital proteins in the innate immune system across species, including humans. This indicates the direct cleanup effect of virus-specific antibodies (Ge et al., 2022). They sought to decipher the role of adaptive immunity elicited by CHIKV/IRES in protection against CHIKV and o‘nyong-nyong virus infection (Chu et al., 2013). Maternal transfer of immunity is crucial for offspring survival (Partidos et al., 2012). Muthumani et al. (2016) demonstrated that administering synthetic DNA-encoded antibodies via injection rapidly provided immunity against CHIKV in mice, effectively neutralized various virus strains *in vitro* and safeguarded mice from lethal infection. Polyclonal and monoclonal antibodies are immunotherapeutics that provide immunity against CHIKV infection. Advances in monoclonal antibody technologies have resulted in diverse antibodies targeting various epitopes of the E1 and E2 envelope glycoproteins (Kim et al., 2019; Segato-Vendrameto et al., 2023). Recent progress in anti-CHIKV monoclonal antibodies, especially those showing efficacy in preclinical models or clinical trials, suggests their potential as a new therapeutic approach (Kim et al., 2019; Segato-Vendrameto et al., 2023). These antibodies, which are directed at different CHIKV epitopes, also contribute to the design of subunit vaccines (Kim et al., 2019; Segato-Vendrameto et al., 2023). Therapeutic monoclonal antibodies (mAbs) offer potential value as alternate therapies for pre- and postexposure protection (Weber et al., 2017). At present, research on mAb-based passive therapy for arboviruses is at an early stage; a few therapeutic studies on CHIKV mAbs have shown that neutralizing antibodies are protective in passive transfer studies and mainly target the CHIKV structural proteins E1/E2 (Weber et al., 2017; da Cruz Silva et al., 2022). Neutralizing antibodies raised against the E2 protein have been shown to protect animal models and murine monoclonal antibodies against E2 (Weber et al., 2017; da Cruz Silva et al., 2022). In addition to mouse monoclonal antibodies, few reports have described the development of human monoclonal antibodies and their role in protection against CHIKV infection. Scientists have also developed Env-specific anti-CHIKV monoclonal antibodies for prophylactic and therapeutic measures against CHIKV infection (Chandley et al., 2023; Segato-Vendrameto et al., 2023). There is enough evidence in the literature regarding immunogenic epitopes recognized by protective monoclonal antibodies. Research has explored the impact of chikungunya and mayaro viruses on cellular immune responses by identifying genes that initiate antiviral pathways, such as Toll-like, RIG-I, and NOD-like receptors, and induce Eotaxin and IL-6, revealing potential molecular targets for antiviral therapies addressing inflammatory responses (Danillo Lucas Alves and Benedito Antonio Lopes da, 2018). Other immune-regulating and pathogenic pathways or responses, such as NFKB, T-cell receptor, TGFβ, MAPK, PI3K-Akt, B-cell receptor, natural killer cell-mediated cytotoxicity, and apoptosis, could serve as potential alternative antiviral targets or biomarkers for CHIKV infection (Uhrlaub et al., 2016; Van Huizen and McInerney, 2020; Islam and Khan, 2021; Mudaliar et al., 2021). This review provides details about the efficacy and safety of current and emerging CHIKV vaccine candidates, including RNA-based vaccines, in addressing the global health threat posed by CHIKV. This study also provides detailed insight into the potential of monoclonal antibodies and other immunotherapies in enhancing protective immunity against CHIKV, considering gaps in the understanding of viral replication, the immune response, and disease management.

## 2 Epidemiology of chikungunya

The CHIKV virus poses a significant health threat to both individuals and their communities. Its impact includes acute symptoms such as arthralgia, rash, fatigue, fever, and myalgia (Gérardin et al., 2008). Regions with a high prevalence of dengue, such as urban centers in Africa and Asia, experience dual outbreaks of CHIKV (Erin Staples et al., 2009). For instance, in Lamu, Kenya, more than 70% of the island’s population was affected by the first outbreak, followed by another outbreak in the Union of Comoros in January 2005, where more than 63% of the population totaled 225,000 (Furuya-Kanamori et al., 2016). India experienced its initial chickenpox outbreak in 1963; the second most significant outbreak occurred in 2006. During the 2006 epidemic, the national burden of CHIKV was estimated to be 25,588 daily, with Karnataka contributing 55% of the national burden (Krishnamoorthy et al., 2009). In 2015, 43.15% of clinically diagnosed dengue patients were positive for the CHIKV virus, surpassing the number of confirmed dengue cases in laboratories (Stewart-Ibarra et al., 2018). CHIKV can impact quality of life by causing postinfection symptoms such as rheumatism, which affects joints and exacerbates preexisting chronic inflammatory rheumatism. In France, 57% of individuals experience rheumatic pain 15 months after CHIKV infection (Sissoko et al., 2009). In 2014, 38 million Americans experienced chronic inflammatory rheumatism (Rodriguez-Morales et al., 2015). During this epidemic, Viremic travelers introduced CHIKV into nonendemic countries, leading to local transmission in several nations, such as Italy, France, New Caledonia, Papua New Guinea, Bhutan, and Yemen (Weibel Galluzzo et al., 2015). Three different genetic forms of the CHIKV have been identified: West African, East/Central/South African (ECSA), and Asian. Since 2004, an epidemic has spread in tropical and subtropical areas worldwide, including Africa, Asia, Europe, the Pacific Islands, and the Americas. The outbreaks are linked to ECSA or Asian genotype viruses, and occasionally both, depending on the region (Petersen and Powers, 2016). CHIKV has emerged as a worldwide health concern over the past two decades. Although its mortality rate is low, there is a high incidence of long-term disability, which poses a significant health risk (Mourad et al., 2022).

## 3 Immunology of chikungunya virus

**Figure 2:**
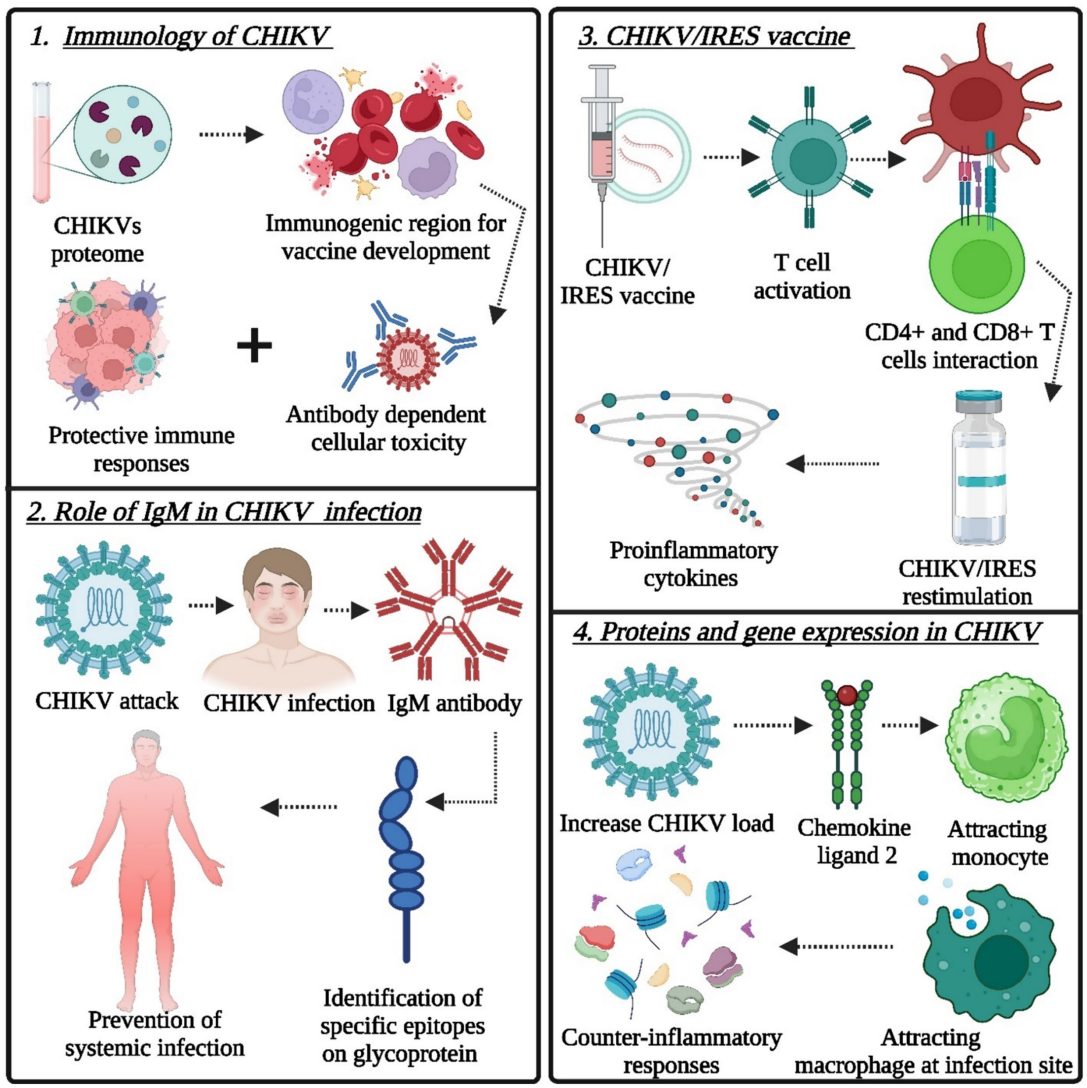
Target points of the immune system for the design and development of novel immunotherapeutics (vaccine technology): (1) role of protective immune responses, (2) role of IgM in CHIKV infection, (3) CHIKV/IRES vaccine for the induction of proinflammatory cytokines after CHIKV/IRES re-stimulation, and (4) proteins and gene expression in CHIKV (Created by using Biorender.com).

A vaccine must trigger both humoral and cell-mediated immune responses to fully prevent reinfection. T-cell epitope-based vaccine design identifies virus-specific immune triggers for targeted vaccine development (Anwar et al., 2019; Jadoon et al., 2019). Immunoinformatics aids in identifying key virus epitopes, expediting research, and saving resources (Priya et al., 2014). The ability of the CHIKV proteome to predict immunogenic regions for vaccine development and potential drug targets for treatment could guide future lab efforts against CHIKV, as presented in Figure 2. The protection afforded by antibodies could be attributed to their capacity to neutralize CHIKV directly and to induce other protective immune responses, such as antibody-dependent and complement-mediated cellular cytotoxicity (Chu et al., 2013; Jin et al., 2015). Effective protection against persistent arthritis in natural CHIKV infection involves crucial contributions from IgG antibodies, particularly IgG3 for neutralization, while IgM complements IgG in immune responses; however, a discrepancy in IgG levels poses a challenge to achieving optimal protection (Patil et al., 2020; Chandley et al., 2023). IgM antibodies offer short-term protection in the early phase, and neutralizing antibodies are vital for preventing symptomatic CHIKV infection by identifying specific epitopes on CHIKV glycoproteins (Verma et al., 2019; Broban et al., 2023). The presence of circulating CD8+ T cells is associated with the acute phase of infection, whereas CD4+ T-cell responses develop at a later stage of infection (Chu et al., 2013). Humoral immunity has been identified as a potential immune correlate of protection against CHIKV infection. Many CHIKV vaccine candidates are currently being developed to generate long-term humoral responses. Clinical trials on these various vaccine candidates have demonstrated the role of humoral immunity in CHIKV infection management (de Lima Cavalcanti et al., 2022). A single dose of the live attenuated CHIKV/IRES vaccine effectively triggered T-cell activation, reaching its peak on the 10th day postimmunization. It induced the production of proinflammatory cytokines (IFN-γ, TNF-α, and IL-2) by memory CD4+ and CD8+ T cells upon CHIKV/IRES restimulation (Chu et al., 2013). Passive immunization with anti-CHIKV/IRES immune serum provided protection, establishing a minimum protective neutralizing antibody titer. The CHIKV/IRES vaccine generates both humoral and cellular immune responses, with humoral immunity being the primary mediator of protection during the acute phase of CHIKV infection, followed by the activation of adaptive immunity (Plante et al., 2011). CHIKV/IRES elicits a strong neutralizing antibody response consisting of all IgG isotypes detected in the serum (Chu et al., 2013). Parida M. M. et al. showed increased activity of specific genes and cytokines and significant upregulation of the TLR3, TRAF-6, TICAM-1, MCP-1, CXCL-10, IL-6, IL-4, ISG-15, MX-2, IFN-β, and OAS-3 genes related to the immune response in mouse brains infected with a virus, resulting in clearance of the virus by days 9–10 (Khan et al., 2012). The use of Poly I:C, a compound that activates immune responses, protected mice from the virus by enhancing the activity of certain genes, suggesting its potential as a preventive treatment against the virus (Salem et al., 2009; Li et al., 2012). Type I IFN is vital for regulating viral replication. Nevertheless, it is not adequate for the full elimination of CHIKV, as the virus persists in tissues even after IFN levels normalize, emphasizing the crucial role of adaptive immunity, where T cells and antibodies, particularly those from memory B cells, play a significant role in providing long-term protection (Rodriguez et al., 2003; Couderc et al., 2008).

**Figure 3:**
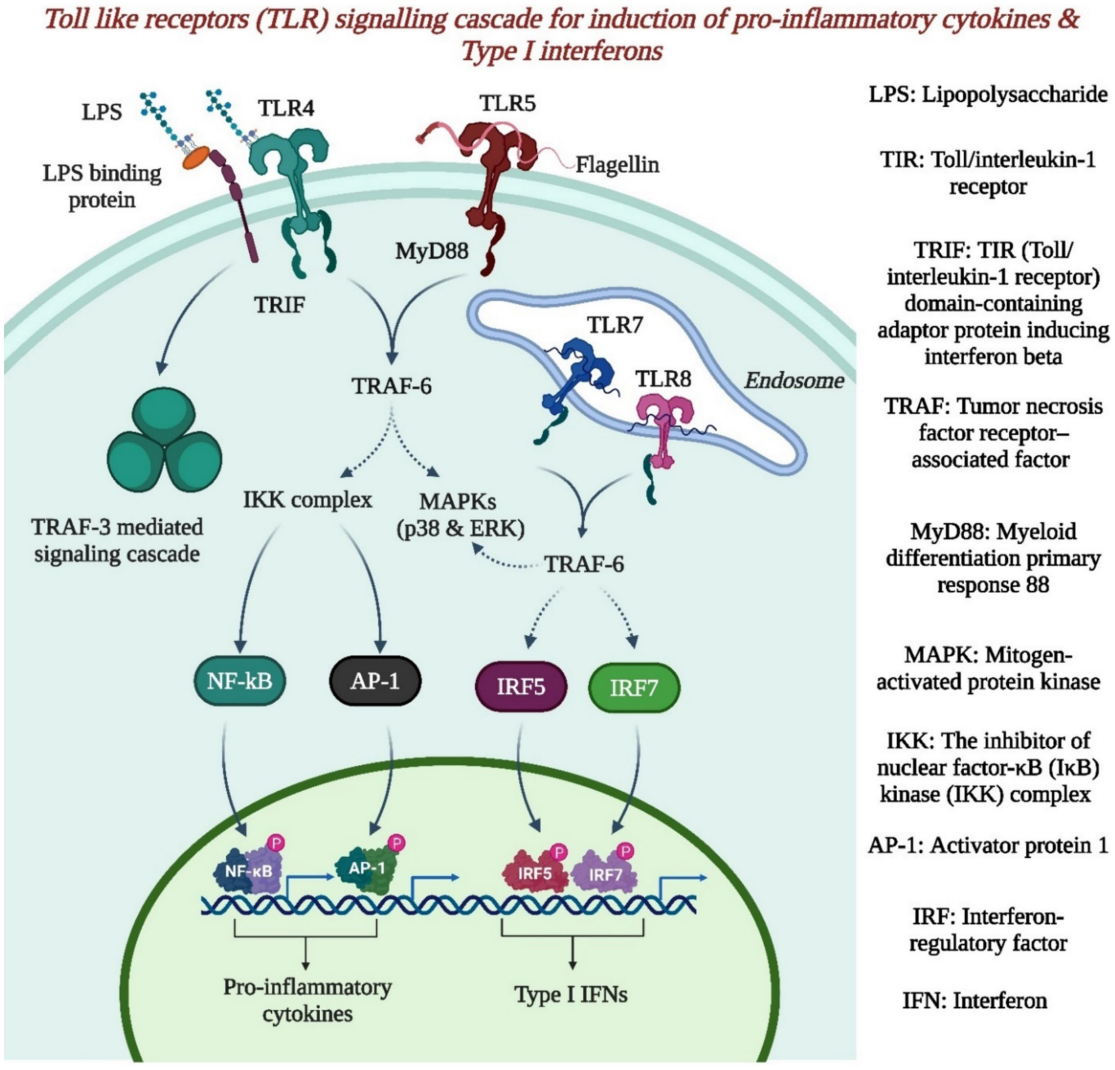
The toll like receptor signaling cascade for induction of proinflammatory cytokines & type 1 interferons (Created by using Biorender.com).

Understanding innate and adaptive immune mechanisms will aid in understanding the pathogenesis of these infections and identifying potential therapies due to the lack of treatments. The depletion of interferon receptors leads to more severe disease in mice, which represents an innate immune response. The expression of interferons, particularly IFN-α, is triggered by various pathways, such as the RIG-I, MDA-5, and TLR pathways, upon Alphavirus infection. However, in the present study, IFN-β remained unchanged, while IFN-α was expressed following MAYV and CHIKV infections (Danillo Lucas Alves and Benedito Antonio Lopes da, 2018). Pattern recognition receptors (PRRs), including TLRs, NOD-like receptors, RIG-I-like receptors, and C-type lectin receptors, are vital components of the innate immune system and are responsible for detecting various pathogen-associated molecular patterns and defending the host organism against bacteria, viruses, and fungi (Brasier et al., 2008; Danillo Lucas Alves and Benedito Antonio Lopes da, 2018; Valdés-López et al., 2022; Gosavi et al., 2023). TLR activation leads to the expression of cytokines and other genes involved in the immune response, as presented in Figure 3. NOD-like receptors lead to the activation of proinflammatory caspases, and RIG-like receptors recognize specific genetic fragments in the cytosol, typically double-stranded RNA or single-stranded RNA (Danillo Lucas Alves and Benedito Antonio Lopes da, 2018; Valdés-López et al., 2022).

**Figure 4:**
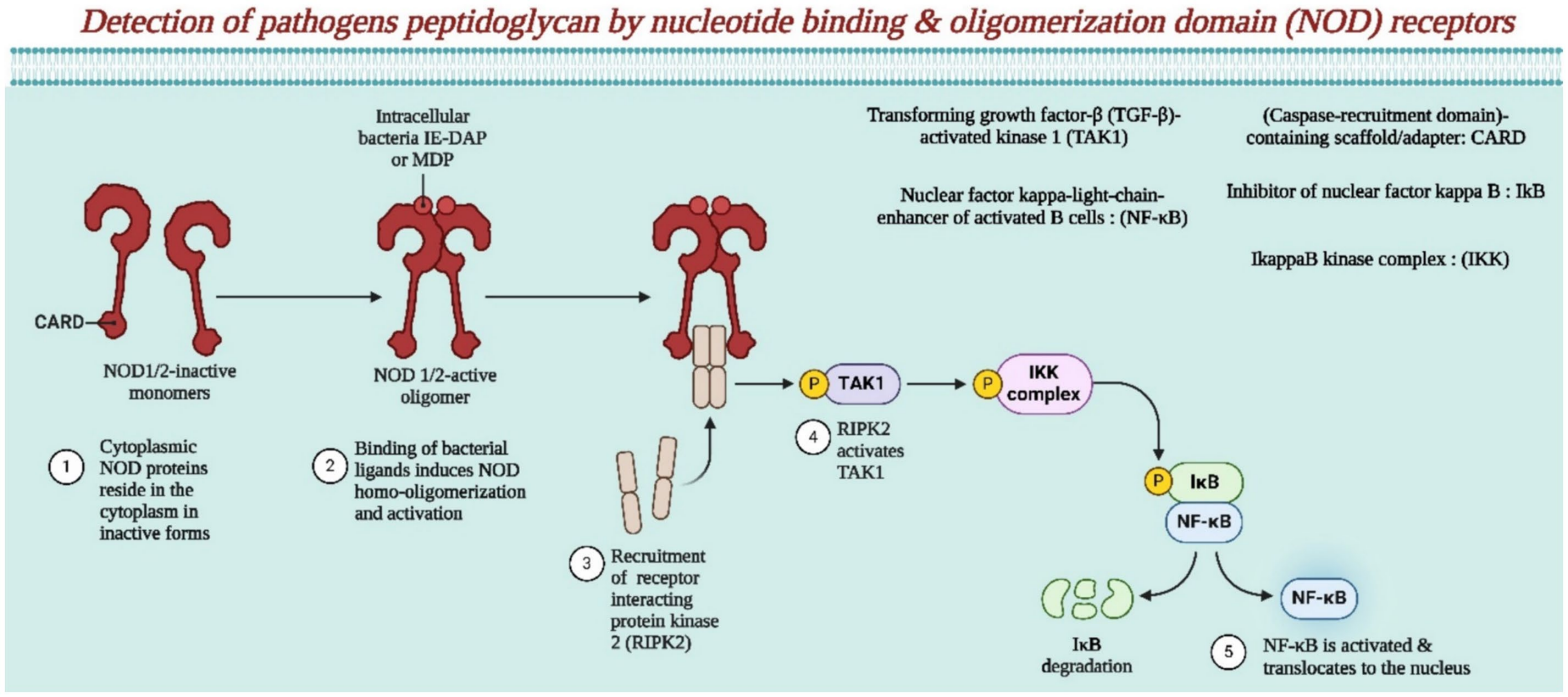
This illustrates the detection of pathogenic peptidoglycan by NOD receptors (Created by using Biorender.com).

The involvement of the NOD receptor in pathogenic peptidoglycan is presented in Figure 4. All three pathways ultimately result in the expression of immune-related genes, such as cytokines, chemokines, interferons, or ISGs (Danillo Lucas Alves and Benedito Antonio Lopes da, 2018). Recently, a cytoplasmic sensor protein (cGAS) was identified that detects double- and single-stranded DNA during viral infections, triggering an interferon type I response (Sun et al., 2013). Additionally, these findings confirm earlier findings of peak CCL-2 and IL-6 levels during peak viral load, highlighting an early innate immune response while indicating consistent local high expression of IFNγ and TNF-α during the incubation and symptomatic phases, suggesting their role in antiviral/proinflammatory responses (Spellberg and Edwards, 2001; Rulli et al., 2009, 2011). Notably, unlike the therapeutic effect seen in IIM, alphavirus infections show increased viral titers and worsened myositis with anti-TNF drugs (Herrero and Mahalingam, 2011; Patil et al., 2012). The mRNA levels of specific chemokines (CCL-2, CXCL-10, and CXCL-11) and their respective receptors (CCR-2 and CXCR-3) increase (Olson and Ley, 2002). This finding points to the importance of CCR-2 in recruiting innate immune cells such as blood monocytes and NK cells during the early peak of viral load (Olson and Ley, 2002). Conversely, CXCR-3 is crucial for attracting CTL and Th1 cells at a later symptomatic stage. In animal models, IFNγ induces CXCL-10 expression and an IL-10 response, indicating potential common inflammatory pathways in acute viral myopathies (Patil et al., 2012). Temperature influences the immune response in *Aedes aegypti* infected with the chikungunya virus. This study revealed that temperature-dependent variations occur in pathways such as Toll, Imd, Jak–Stat, siRNA, and apoptosis pathways, indicating the modulation of innate immunity during CHIKV infection in *Aedes aegypti* (Wimalasiri-Yapa et al., 2020). During the peak of the viral load, specific immune signals showed a notable increase, indicating a Th1 response. This response involved heightened activity of specific molecules and cells, such as CXCR-3, TBX-21, and IFNγ, as well as elevated levels of cytokines such as IL-2, IFNγ, and IL-17 (Chow et al., 2011; Tanabe et al., 2019). Interestingly, the presence of specific antibodies supported this immune reaction. Additionally, the study revealed that NK cells play a significant role in boosting IFNγ levels early on, while T cells join the immune response later. The levels of specific inflammatory markers increase throughout the illness course, indicating their pivotal role in chikungunya progression (Arankalle et al., 2015; Babu et al., 2023). The balance between pro- and anti-inflammatory molecules appears to be crucial in determining disease severity. Notably, the expression of a key chemokine, CCL-3, which is important for activating CD8+ T cells, peaked during the symptomatic phase, suggesting its involvement in the adaptive immune response (de Sousa Dias et al., 2018; Davenport et al., 2020; Mapalagamage et al., 2022).

The highest levels of certain proteins (CCL-2, KC, CCL-4, RANTES, IL-6, IL-10, CSF-3) in the bloodstream and gene expression (CCL-2, CXCL-10, CXCL-11) along with IFNγ, IL-10, STAT-1, SOCS-1, and CSF-3 suggest a strong adaptive immune response during the peak viral load (Patil et al., 2012). When symptoms appeared, there were elevated levels of IL-2, IFNγ, IL-17, CCL-3, IL-1b, eotaxin, IL-9, and CSF-2 in the blood and an increase in the expression of genes linked to proinflammatory responses in the affected tissues, mainly favoring Th1 immune cells (Khan et al., 2012; Patil et al., 2012). The levels of chemokines such as CXCL-10, CXCL-11, CCL-2, and CCL-5 initially increased but decreased significantly during symptom onset. CCL-3 peaked during symptoms. Receptors such as CXCR-3 and CCR-2 were upregulated during incubation, with CCR-2 peaking during this phase and CXCR-3 peaking during symptoms (Patil et al., 2012; Reddy et al., 2014; Tanabe et al., 2019). The increased expression of T-cell and macrophage surface markers aligned with increased CXCR-3 and CCR-2 levels, suggesting their role in immune cell movement to the infection site (Patil et al., 2012; Reddy et al., 2014).

Th1 cytokines such as IFN-γ peaked during incubation and remained steady throughout the symptomatic period. Additionally, the levels of IL-2 and TBX-21, which are crucial for CD4 cell commitment to Th1 cells, peaked during symptoms. The levels of IL-15, IL-18, and IL-12, other Th1-stimulating cytokines, peak early and decrease sharply during the symptomatic phase (Ng et al., 2009; Patil et al., 2012; Restrepo et al., 2022). (6-II) During the incubation phase, there was a high level of Th2 cytokines (IL-4, IL-10, and IL-6), which decreased significantly when symptoms appeared (Ng et al., 2009; Patil et al., 2012; Liu et al., 2022; Restrepo et al., 2022). During the symptomatic phase, the peak expression of proinflammatory cytokines, such as NoS-2 (iNoS), TNF-α, Il-1α, IL-1β, COX-2 (PTGS-2), and CCL-3, significantly increased (Patil et al., 2012; Chirathaworn et al., 2020; McCarthy et al., 2020). Cell surface antigens such as CD3, CD4, CD8, ICOS, CD40Lg, H2Ea, and H2Eb1 were notably increased during the symptomatic phase compared to the incubation period, indicating a surge in T-cell presence (Patil et al., 2012; Teo et al., 2018; Gois et al., 2022). The MHC class II marker H2Eb1 showed higher levels during recovery than in the symptomatic phase, while other T-cell antigens decreased slightly during recovery (Patil et al., 2012; Kori et al., 2015).

A recent study revealed that during peak viral load (day 3 PI), there were high levels of CCL-2, IL-6, and IL-10 locally and in the bloodstream, similar to what was observed in human studies. In mouse models, CCL-2 seems to play a role in attracting immune cells (monocytes/macrophages) to the infection site (Rulli et al., 2009, 2011). Additionally, there was an increase in keratinocyte chemoattractant (KC), similar to human IL-8, at peak viral load (Mateo et al., 2000). These findings suggest that during peak virus levels, a response triggers both inflammatory and counterinflammatory responses to IFNγ, with IL-10 likely playing an essential immunomodulatory role (Patil et al., 2012). The identification of CXCL-10 as a potential biomarker for CHIKV severity in humans and its relevance as a drug target emphasize the usefulness of a mouse model for studying CHIKV infection (Ninla-Aesong et al., 2019; Tanabe et al., 2019; Chirathaworn et al., 2020). Additionally, the results indicate that a strong IFNγ program is associated with the upregulation of specific genes during peak virus loading. This activation of the STAT-1 pathway differs across diseases such as rheumatoid arthritis, inflammatory myopathies, and osteoarthritis due to variations in IFN expression (Gallardo, 1999; De Hooge et al., 2004; Kasperkovitz et al., 2004). Elevated CD3+ CD8+ T cells in the early disease stages suggest a role for activated cytotoxic T lymphocytes in clearing CHIKV-infected cells. This study suggested tissue-specific functions for CD40L expression and highlighted the potential influence of the CD40-CD40L interaction on the immune response (Mach et al., 1997; Sugiura et al., 2000; Wauquier et al., 2011). The suspected involvement of activated T cells in skeletal muscles in idiopathic inflammatory myopathies has also been discussed (Dhanwani et al., 2014; Muelas et al., 2017).

**Figure 5:**
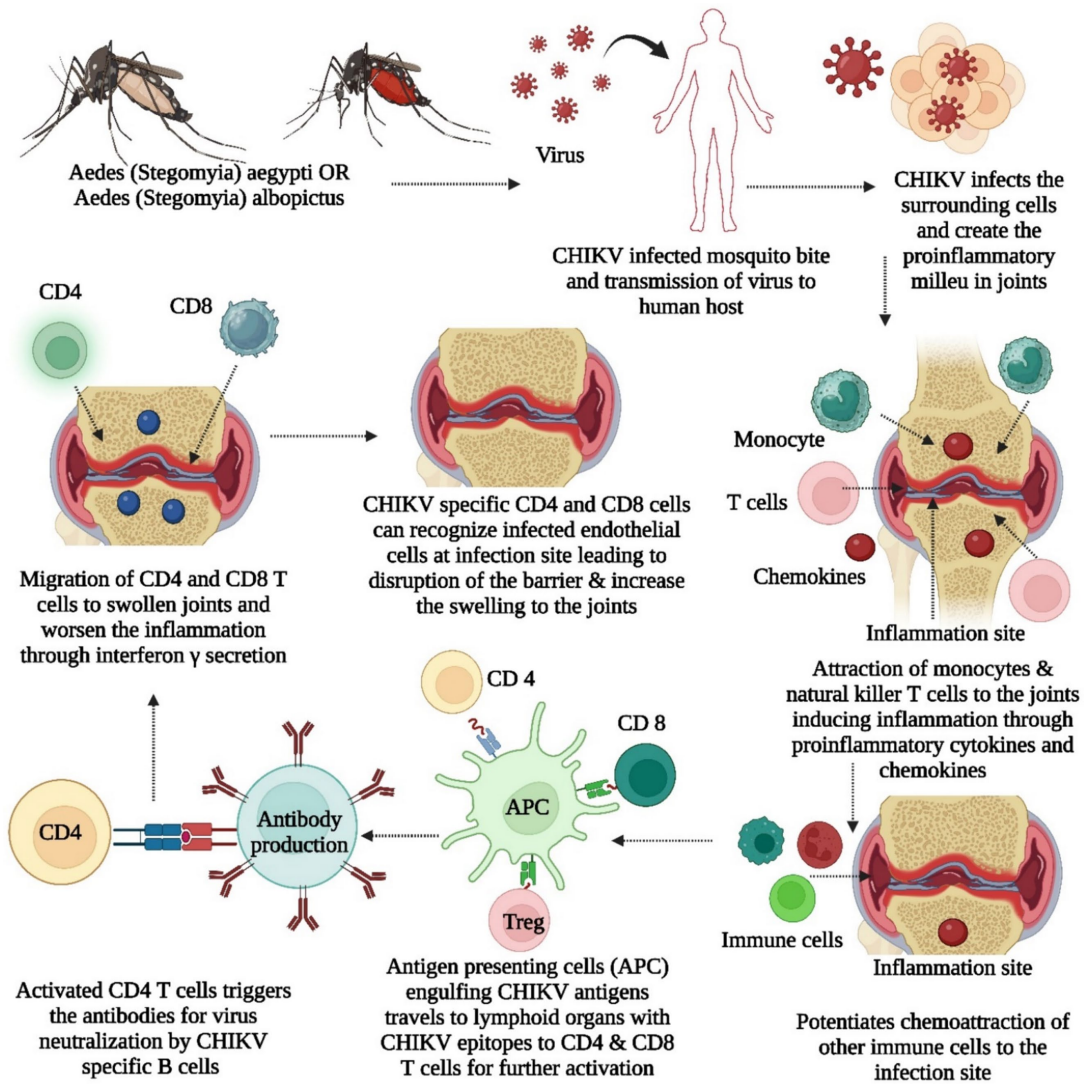
It illustrates about the role of CD4 and CD8 cells into CHIKV, contribution of proinflammatory and inflammatory chemokines, cytokines, antigen presenting cells for induction of swelling and inflammation.

The virus multiplies within cells, generating new viral proteins, with MHC class I presenting antigens to T CD8+ cells for targeted cell destruction and MHC class II activating T CD4+ cells influencing immune responses through cytokines, controlling infection and inhibiting viral replication (Kori et al., 2015; Heath et al., 2016; de Sousa Dias et al., 2018). Research indicates the importance of molecular mimicry in CHIKV-induced arthritis, revealing immune epitopes shared with human proteins linked to arthritis, such as FLT1, KDR, TIE1, PADI4, FCRL3, PTPN22, and CSK, and suggesting that antibodies targeting these epitopes may contribute to autoimmune responses in CHIKV infection, particularly in the context of arthritis, urging further exploration with suitable animal models (Ding et al., 2021). Mario Perkovic et al. developed a CHIKV vaccine with trans-amplifying RNA (taRNA), which is composed of two RNAs. One is a nonreplicating mRNA encoding CHIKV nonstructural proteins, and the other is a trans-replicon (TR) RNA encoding CHIKV envelope proteins (Schmidt et al., 2022a,b). When amplified by the replicase, the TR-RNA induces a robust immune response with high protein expression. The vaccine elicited strong CHIKV-specific immune responses in a mouse model and protected against high-dose CHIKV challenge infection. taRNAs are a promising and safe vaccination strategy for CHIKV infections (Schmidt et al., 2022a). Depleting IFN-γ-producing CD4+ T cells reduces joint swelling in CHIKV-infected mice while the role of CD4 and CD8 cells in CHIKV are illustrated in Figure 5. Nevertheless, both CD4+ and CD8+ T cells are crucial for the efficacy of a cytotoxic T lymphocyte (CTL)-based vaccine against CHIKV, highlighting the challenge of developing a comprehensive vaccine that induces protective antibodies and appropriate T-cell responses (Burrack et al., 2015; Broeckel et al., 2019; Ge et al., 2022). Recent scientific research indicates that mRNA vaccines have the potential to effectively combat viruses such as HIV, Zika virus, dengue virus, and influenza virus by inducing robust cellular and humoral immune responses without the need for adjuvants (Pollard et al., 2013; Bahl et al., 2017; Richner et al., 2017; Vogel et al., 2018; Zhang et al., 2020).

## 4 Chikungunya vaccine landscape and IXCHIQ vaccine development

Alphaviruses, including CHIKV, have been under vaccine development for years, with CHIKV vaccines progressing from preclinical stages to promising clinical trial assessments (Ramsauer and Tangy, 2016; Powers, 2018). The genomic structure of the virus, which includes different lineages, has been identified through whole-genome sequencing. Complete CHIKV genome sequencing revealed the existence of four lineages: West African (Waf), East/Central/South African (ECSA), Asian, and Indian Ocean Lineage (IOL) (Volk et al., 2010). The historical context highlights the ongoing need to address the changing nature of the virus, leading to the development of vaccines that show promise in clinical trials. The IXCHIQ vaccine, which was first approved by the US FDA, is specifically designed to target common antigens of the CHIKV virus found in various strains (Idse et al., 2023). Efforts are underway to develop a vaccine for the disease CHIKV. Various types of vaccines, such as live-attenuated, virus-like particle, and mRNA vaccines, are being tested to determine their safety and effectiveness in preventing CHIKV infection (Akahata et al., 2010; Chang et al., 2014; Ramsauer et al., 2015; Roongaraya and Boonyasuppayakorn, 2023).

The development of a CHIKV vaccine faces obstacles such as the genetic diversity of the virus, making it challenging to develop a vaccine that covers various strains. Research in Mexico and Brazil revealed numerous mutations and distinct lineages, complicating vaccine design (Rodríguez-Aguilar et al., 2022). During the CHIKV outbreak in Mexico from 2014 to 2016, extensive genetic variability was observed, revealing 70 nonsynonymous mutations in the NSP3, E1, and E2 genes (Rodríguez-Aguilar et al., 2022; Hakim and Aman, 2023). Understanding the spread dynamics and evolutionary history is crucial for developing effective vaccines that can combat diverse strains of CHIKV (Matusali et al., 2019; Simon et al., 2023). The complex immune responses to CHIKV, safety concerns, regulatory hurdles, and nature of the virus RNA further complicate vaccine development (Shaw et al., 2019; Menon and Wilder-Smith, 2023). Limited understanding of the long-term immune response needed, high costs, resource limitations, and challenges in clinical trials add to the difficulties (Hakim and Aman, 2023; Menon and Wilder-Smith, 2023; Tran et al., 2023). Ensuring the safety of CHIKV vaccines, especially in vulnerable populations, is a crucial aspect of vaccine research. Global focus and market forces tend to prioritize diseases affecting wealthier or larger populations, leading to more attention and funding than those impacting developing regions, such as CHIKV in tropical areas (Menon and Wilder-Smith, 2023). Despite these challenges, ongoing efforts are aimed at overcoming these obstacles and providing a safe and effective CHIKV vaccine to the market, with promising candidates in preclinical and early clinical stages (Shaw et al., 2019). Vaccine development opportunities arise from advancements in platforms such as mRNA and viral vectors, which offer innovative avenues for CHIKV vaccine research, as shown in Table 1 (Harrison et al., 1971; Shaw et al., 2019). An increased understanding of immune responses to infection allows for the design of vaccines that induce solid and lasting protective immunity. The global collaboration among researchers and organizations and the potential public health impact of a CHIKV vaccine.

**Table 1:** CHIKV vaccine clinical trials are registered on the following websites: https://www.clinicaltrials.gov and https://www.anzctr.org.au.

Vaccine candidate	Clinical trial status	Intervention (Biological – B, Drug – D)	Sponsored by	Collaborator	Clinical trial registration no.	Study Status	Sex	Age	Phase	Enrollment	Study design Allocation: na intervention model	Study type	References
Pxvx0317	Completed	Biological – Chikv Vlp, Adjuvanted	Bavarian Nordic	Emergent Biosolutions	Nct05065983	Completed	All	Adult	2	25	Single – Group Masking – None (Primary Purpose – Prevention)	Interventional	https://clinicaltrials.gov/study/NCT05065983?intr=Nct05065983&rank=1
Pxvx031	Recruiting	Biological – Pxvx0317 Vaccine Booster & Placebo Booster	Bavarian Nordic		Nct06007183	Recruiting	All	Child, Adult, Older Adult	3	800	Parallel – Masking: Triple (Participant, Care Provider, Investigator) (Primary Purpose – Prevention)	Interventional	https://clinicaltrials.gov/study/NCT06007183?intr=PXVX0317%20vaccine%20booster&rank=1
Live-Attenuated Chikungunya Virus Vaccine	Completed	Biological – Vla1553 & Placebo	Valneva Austria Gmbh		Nct04546724	Completed	All	Adult, Older Adult	3 3	4,128	Parallel – Masking: Double (Participant, Investigator) (Primary Purpose – Prevention)	Interventional	https://clinicaltrials.gov/study/NCT04546724?intr=Nct04546724&rank=1
Vla1553	Not Yet Recruiting	Biological – Vla1553	Valneva Austria		Nct06028841	Not Yet Recruiting	All	Adult, Older Adult	3	75	Single – Group Masking – None (Primary Purpose – Prevention)	Interventional	https://clinicaltrials.gov/study/NCT06028841?intr=Nct06028841&rank=1
Chikungunya Vaccine	Active Not Recruiting	Drug – Bbv87 Chikungunya Vaccine & Normal Saline	International Vaccine Institute		Nct04566484	Active Not Recruiting	All	Child, Adult, Older Adult	2	3,210	Sequential – Masking Double (Participant, Investigator) (Primary Purpose – Prevention)	Interventional	https://clinicaltrials.gov/study/NCT04566484?intr=Nct04566484&rank=1
Live-Attenuated Chikungunya Virus Vaccine	Completed	Biological – Biological Vaccine Vla1553	Valneva Austria Gmbh		Nct04786444	Completed	All	Adult	3	409	Parallel – Masking: Double (Participant, Investigator) (Primary Purpose –Prevention)	Interventional	https://clinicaltrials.gov/study/NCT04786444?intr=Nct04786444&rank=1
Chikungunya And Zika Vaccines	Completed	Biological – Chik Low Dose, Chik Mid Dose, Chik High Dose & Zika Low Dose, Zika Mid Dose, Zika High Dose & Saline Placebo	University Of Oxford		Nct04440774	Completed	All	Adult	1 1	120	Sequential – Masking – Double (Participant, Care_Provider) (Primary Purpose – Prevention)	Interventional	https://clinicaltrials.gov/study/NCT04440774?intr=Nct04440774&rank=1
Vla1553	Not Yet Recruiting	Biological – Vla1553 Full Dose, Vla1553 Half Dose & Control	Valneva Austria Gmbh		Nct06106581	Not Yet Recruiting	All	Child	2	300	Parallel – Masking – Quadruple (Participant, Care Provider, Investigator, Outcomes Assessor) (Primary Purpose – Prevention)	Interventional	https://clinicaltrials.gov/study/NCT06106581?intr=Nct06106581&rank=1
Live-Attenuated Chikungunya Vaccine	Not Yet Recruiting	Biological – Active & Placebo	Butantan Institute	Valneva Austria Gmbh	Nct04650399	Active Not Recruiting		Child	3	750	Parallel – Masking: Double (Participant, Investigator), (Primary Purpose – Prevention)	Interventional	https://clinicaltrials.gov/study/NCT04650399?intr=Nct04650399&rank=1
Chikungunya Vaccine, Pxvx0317 Chikv-Vlp	Completed	Biological – Chikv Vlp / Unadjuvanted, Chikv Vlp /Adjuvanted & Placebo	Emergent Biosolutions	Bavarian Nordic	Nct03483961	Completed		Adult	2	445	Parallel – Masking – Quadruple (Participant, Care Provider, Investigator, Outcomes Assessor) (Primary Purpose – Prevention)	Interventional	https://clinicaltrials.gov/study/NCT03483961?intr=Nct03483961&rank=1
Vla1553	Not Yet Recruiting	Biological – Vla1553		Valneva Austria Gmbh	Nct04838444	Active Not Recruiting		Adult, Older Adult	3	363	Single – Group Masking – None (Primary Purpose – Prevention)	Interventional	https://clinicaltrials.gov/study/NCT04838444?intr=Nct04838444&rank=1

IXCHIQ shares antigens across different virus strains, offering broad-spectrum protection (Chikungunya Vaccine, 2023). Preclinical studies indicate that IxChiq can induce a robust and lasting immune response with an acceptable safety profile (Clinical Trials Arena, 2023). Given the urgent need for an effective CHIKV vaccine and the global impact of outbreaks, IxChiq has emerged as a promising candidate for further development.

### 4.1 Clinical studies

The efficacy of IXCHIQ relies on immune response data from a U.S. clinical study involving individuals aged 18 and above. The study compared the immune responses of 266 vaccinated participants with 96 who received a placebo. The antibodies assessed were determined to be protective in nonhuman primates, and almost all vaccine recipients reached this protective antibody level in the study. After reconstitution, a clinical study was conducted by administering a 0.5 mL single dose of IXCHIQ through intramuscular injection. Pregnancies carry inherent risks, such as congenital disabilities and miscarriage. No sufficient or well-regulated studies on IXCHIQ in pregnant individuals exist, and the limited human data from clinical trials are insufficient to determine potential risks during pregnancy. However, a rat study in which a single human dose of IXCHIQ was administered before mating and during gestation revealed no harm to the fetus or adverse effects on postnatal development (Idse et al., 2023; Robert and Brown, 2023).

The safety of the IXCHIQ was assessed in two clinical studies involving 3,490 participants aged 18 and older in North America. Study 1, a randomized, double-blinded, placebo-controlled trial, included 3,082 participants receiving IXCHIQ and 1,033 receiving a placebo. Study 2, a non-placebo-controlled study, involved 408 participants receiving the IXCHIQ. Among the 4,523 participants across both studies, 54.7% were females, 80.1% were White people, 14.0% were Black people, 1.9% were Asians, and 17.2% were Hispanics or Latinos. Solicited adverse reactions were recorded for the first ten days postvaccination, and unsolicited adverse events were monitored for up to 6 months postvaccination. Hematology parameters were assessed at specific intervals in a subset of participants from Study 1. These findings underscore the need for caution in comparing adverse reaction rates between different vaccines and highlight the diverse demographic characteristics of the studies (Robert and Brown, 2023).

### 4.2 Preclinical studies

In a study involving female rats, a complete human dose of IXCHIQ (0.5 mL) was given through intramuscular injection twice – 14 days before mating and on gestation day 6. This study aimed to assess the impact on female fertility, reproductive performance, and prenatal and postnatal development. No adverse effects related to the vaccine were observed on fetal development, reproductive performance, or prenatal or postnatal development (Park, 2010; Robert and Brown, 2023).

### 4.3 Animal toxicology and pharmacology

In a study involving nonhuman primates (NHPs), human anti-CHIKV immune sera from a phase 1 study (NCT03382964) were passively transferred. In the phase 1 study, a single dose of a vaccine with attenuated CHIKV was administered, generating 8 serum pools with varying anti-CHIKV neutralizing antibody titers from days 14 to 180 postvaccination. In the passive transfer study, 40 CHIKV-naïve cynomolgus macaques received human anti-CHIKV immune sera (n = 5 per group), while six macaques received nonimmune control sera. One day posttransfer, serum samples were collected to determine prechallenge antibody titers. Macaques were then challenged with wild-type CHIKV, and monitoring included assessing viremia and body temperature. Animals receiving postvaccination serum showed no fever postchallenge, while those receiving nonimmune serum exhibited fever and viremia. An analysis indicated that a μPRNT50 titer of ≥150 was likely to predict clinical benefit in a phase 3 study (Gérardin et al., 2008; Park, 2010; Robert and Brown, 2023).

## 5 Different vaccine platforms and technologies used in CHIKV vaccine development

### 5.1 Formalin-inactivated vaccines (FIV)

CHIKV vaccines were initially developed in the late 1960s using a formalin-inactivated approach at the Walter Reed Army Institute of Research. This study tested vaccines derived from the African CHIKV strain 168 on mice and rhesus macaques. Different cell preparations were used, such as chik-embryo, suckling-mouse-brain, and green monkey kidney cells. The green monkey kidney cell preparation, which was chosen for its safety, showed good immunogenicity. The CHIKV 168 vaccine and CHIK 15562 provided homologous protection in mice, leading to the evaluation of heterologous protection in macaques using strains from Africa, Asia, and India. Vaccinated macaques demonstrated protection against homologous and heterologous challenges, marking the first successful demonstration of protective efficacy of a formalin-inactivated CHIKV vaccine in preclinical models (Harrison et al., 1971; Kumar et al., 2012).

#### 5.1.1 Advantages and challenges

FIV for CHIKV treatment has proven effective in preclinical models, a well-established research history since the late 1960s, and the ability to choose suitable strains and diverse cell preparations. They demonstrate both homologous and heterologous protection, making them versatile. However, challenges include poor immunogenicity in specific preparations such as chick embryos, safety concerns with suckling mouse brain preparations, development complexity due to varied cell preparations, and the resource-intensive preclinical testing process. Transitioning to clinical testing is crucial for validating safety and efficacy in humans but adds complexity and resource demands (Harrison et al., 1971; Holzer et al., 2011).

### 5.2 CHIKV live-attenuated vaccines (LAV)

Initially, researchers looked into formalin-inactivated CHIKV vaccines, but concerns over safety and cost led to the development of a live attenuated CHIKV vaccine in the 1980s. This vaccine, called CHIK 181/clone 25, underwent multiple passages and attenuation processes, proving its effectiveness in protecting mice and rhesus macaques in preclinical trials. Manufactured at The Salk Institute-Government Services Division (TSI-GSD), it entered clinical trials in 1986, demonstrating its safety and immunogenicity in phases I and II. Notably, 98% of vaccinated individuals developed neutralizing antibodies, and a small percentage experienced transient arthralgia. Despite the success of live-attenuated vaccines (CHIK 181/clone 25 & CHIKV TSI-GSD-218), challenges such as biosafety concerns and adverse events have led to the exploration of alternative platforms, such as subunit vaccines, virus-like particles (VLPs), replication-deficient viral vectors, DNA vaccines, and proteins, which are considered safer but may require adjuvants or multiple doses for enhanced effectiveness. Despite these challenges, the persistence of CHIKV outbreaks has attracted increasing interest, and efforts to explore diverse vaccine platforms are ongoing (Gao et al., 2019; Abeyratne et al., 2020; Voigt et al., 2021).

#### 5.2.1 Advantages and challenges

Traditional methods raise concerns regarding cost and safety, necessitating biosafety level 3 facilities. Newer vaccine platforms, though safer and cost-effective, may require adjuvants or multiple doses for optimal immunity. The development of live attenuated CHIKV vaccines involves specific cloning, resulting in complete protection in animal models. Notably, the CHIKV 181/clone 25 vaccine provides sterile protection in macaques, and its derivative, the CHIKV TSI-GSD-218 vaccine, has demonstrated safety and immunogenicity in human trials. Challenges associated with traditional methods have led to the exploration of alternatives such as subunit vaccines and viral vectors, which offer safety advantages despite potentially lower immunogenicity. Ongoing interest in vaccine development is fueled by intermittent CHIKV outbreaks, prompting continuous assessment of new platforms to address evolving challenges (Masrinoul et al., 2014; Voigt et al., 2021; Schneider et al., 2023).

### 5.3 Virus-like particles (VLPs)

VLPs imitate the outer shell of a virus using viral structural proteins (capsid, E3, E2, 6 K, and E1) but lack the genetic material needed for replication, ensuring their safety in vaccines. The Vaccine Research Center (VRC) at the National Institute of Allergy and Infectious Diseases (NIAID) has developed a CHIKV VLP, VRC-CHKVLP059-00-VP, composed of structural proteins from the CHIKV strain 37,997 Waf lineage. In preclinical studies with macaques, the vaccine showed immunogenicity, generating immune responses and enabling the control of CHIKV challenges. Produced under Good Manufacturing Practice, the VLPs were tested in a phase I clinical trial and demonstrated to be safe and tolerable in 25 adults. ELISA revealed positive antibody responses at all doses, with neutralizing antibodies induced after the first vaccination. Following phase I success, phase II trials involving 400 healthy adults across multiple locations aimed to further evaluate the safety and immunogenicity of CHIKV VLPs (Chen et al., 2020; Thompson et al., 2022).

#### 5.3.1 Advantages and challenges

VLPs offer benefits and challenges in vaccine development. They are safe, lack genetic material for replication, and mimic the virus’s structure, prompting a robust immune response. The CHIKV VLP vaccine demonstrates immunogenicity in macaques, controlling viraemia and inflammation. The VLP vaccine (VRC-CHKVLP059-00-VP), which was produced by Good Manufacturing Practices, maintains high quality. Phase I trials confirm safety, tolerability, and positive antibody responses with no serious adverse events reported, leading to phase II trials for broader safety and immunogenicity (Metz et al., 2013; Arévalo et al., 2019; Chen et al., 2020).

### 5.4 Messenger RNA (mRNA) vaccines

mRNA vaccines, a cutting-edge advancement in vaccine technology, offer a rapid development process by utilizing the genetic code of pathogens. Unlike traditional vaccines, which can take months or years, mRNA vaccines instruct the body to produce a virus-specific protein, generating an immune response without causing illness. This adaptable technology allows quick formulation adjustments to target new antigens, enabling the rapid production of high-quality vaccine material. The Pfizer-BioNTech COVID-19 (BNT162b2) vaccine is a notable example of this powerful technique (Shaw et al., 2019; Ge et al., 2022; Jaan et al., 2022; Liu et al., 2023).

### 5.5 Viral-vectored vaccines (VVVs)

#### 5.5.1 Measles viral vector

Developed at the Institut Pasteur in Paris under Frédéric Tangy’s leadership serves as the basis for Viral-Vectored Vaccines (VVV). In 2013, Samantha Brandler and team created a measles virus-vectored (MVV) vaccine expressing CHIKV structural genes. The vaccine exhibited positive results in mice, demonstrating protection against CHIKV in a phase I clinical trial with 42 participants. Subsequent phase 2 trials involving 263 participants showed that both low and high doses of the MV-CHIKV vaccine induced neutralizing antibodies with excellent safety and tolerability, expanding beyond CHIKV, which has applications including vaccines for Ebola and COVID-19 (Astra Zeneca and Johnson & Johnson) (Reisinger et al., 2018; Gerke et al., 2019).

##### 5.5.1.1 Advantages and challenges

Measles viral vector vaccines promise to generate robust immune responses against CHIKV infection with reasonable safety. However, challenges include determining the ideal vaccine dose and schedule, potentially relying on a booster for full effectiveness (100% seroconversion), and assessing long-term efficacy (Ramsauer and Tangy, 2016; Reisinger et al., 2018; Gerke et al., 2019).

#### 5.5.2 Adenoviral vectors

ChAdOx1-Chik vaccines utilizing a chimpanzee adenoviral vector are being explored to overcome challenges associated with preexisting immunity to human adenovirus. The ChAdOx1-Chik vaccine has entered clinical trials to assess its safety, immunogenicity, and efficacy against various CHIKV lineages. This finding underscores the significance of establishing a correlate of protection against CHIKV infection, suggesting a potential role for IgG antibodies in clearing the virus (Kroon Campos et al., 2020; Folegatti et al., 2022).

##### 5.5.2.1 Advantages and challenges

Adenoviral vector vaccines, such as ChAdOx1, have benefits such as overcoming preexisting immunity and generating solid immune responses without adjuvants. They show promise for cross-protection against various isolates, particularly for CHIKV vaccines. However, challenges include the potential reduction in vaccine efficiency due to preexisting immunity to human adenovirus and the need to identify a protection indicator for CHIKV infection (Kroon Campos et al., 2020; Folegatti et al., 2022).

## 6 Clinical trial data and safety profile

As IXCHIQ contains a live, weakened version of the virus, it may cause symptoms that mimic an actual infection, and headache, fever, fatigue, joint and muscle pain, nausea, and tenderness at injection were reported as side effects of the IXCHIQ vaccine when two clinical studies of 3,500 participants aged 18 years and older were conducted in North America to evaluate the safety of this vaccine. In one of these studies, 1,000 participants received a placebo (Table 2). Approximately 1.6% of vaccine recipients developed severe chikungunya-like ADRs that prevented daily activities and required medical intervention, and of these, two recipients were hospitalized. Some recipients had prolonged chikungunya-like ADRs lasting at least 30 days (Park, 2010; Chikungunya Vaccine, 2023; Lucey, 2023).

- To evaluate the effectiveness of the IXCHIQ, the immune response of 266 participants who received the vaccine was compared with that of placebo-treated participants (96) in a clinical study conducted in the USA in individuals 18 years of age and older (Park, 2010; Chikungunya Vaccine, 2023; Lucey, 2023).

**Table 2:** Clinical trial data and safety profile of the live attenuated IXCHIQ vaccine.

Study	Participants	Vaccine recipients	Placebo recipients	Seroresponse rate (28 days postvaccination)	Seroresponse rate (6 months postvaccination)
1	3,500	2,500	1,000	98.9%	96.3%
2	3,500	2,500	1,000	98.9%	96.3%

FDA Approves First Vaccine to Prevent Disease Caused by Chikungunya Virus | FDA

- The vaccine demonstrated a 98.9% seroresponse rate 28 days postvaccination. This seroresponse was sustained over time, with a 96.3% seroresponse rate observed six months postvaccination.
- In a placebo-controlled trial, 98.9% of vaccine recipients met the seroresponse threshold, compared to 0% of the placebo recipients (Park, 2010; Chikungunya Vaccine, 2023; Lucey, 2023).

For the efficacy of the IXCHIQ vaccine, the data from these studies suggested high seroconversion rates and robust generation of neutralizing antibodies following vaccination. This high level of efficacy was observed across different geographies and demographics, highlighting the broad applicability of the vaccine. Regarding safety, findings revealed that the vaccine was generally well tolerated, with most adverse events being mild to moderate, such as injection site pain, mild fever, and temporary fatigue (Park, 2010; Chikungunya Vaccine, 2023; Lucey, 2023). The IXCHIQ vaccine shows high efficacy and a favorable safety profile (Table 3; Cameron, 2023; Chikungunya Vaccine, 2023; Dunleavy, 2023; Idse et al., 2023).

**Table 3:** The US FDA’s approval of Valneva’s IXCHIQ brought the world’s first chikungunya vaccine to market—Clinical Trials Arena.

Phase	Focus	Key findings
Phase I	Safety and immunogenicity	The Phase I trials were conducted on a few healthy volunteers, and the findings are not publicly available.
Phase II	Safety and immunogenicity	In the Phase II trials, a single dose of IxChiq, a live attenuated vaccine, developed viral resistance in 98% of those tested after 28 days, and 85% still showed resistance after one year. However, 8% of people reported transient joint pain.
Phase III	Safety and efficacy	In the pivotal Phase III data reported in 2022, IxChiq demonstrated a 98.9% (263 of 266 subjects tested for immunogenicity) seroresponse rate at 28 days with a single vaccination. 233 of 242 subjects tested for immunogenicity, seroresponse rates at 28, 84, and 180 days postvaccination were 98.9, 98.0, and 96.3%, respectively.

## 7 Public health implications and regulatory considerations

Creating a safe and efficient vaccine for the CHIKV virus is essential, as the disease disrupts life by causing increased illness, school and work absenteeism, and financial strain (Bartholomeeusen et al., 2023). CHIKV can adjust to a different mosquito vector, as noted in Indian Ocean epidemics (Tsetsarkin et al., 2016). Compared with those in mosquitoes at higher latitudes such as *A. aegypti*, mutations in *A. albopictus* enhance infection and contribute to outbreaks in new regions, such as Europe (Rezza et al., 2007). CHIKV infection typically has a low fatality rate, but in impoverished nations such as Mauritius, it reaches 2.3 per 1,000 people, suggesting increased severity (Ramchurn et al., 2008). Outbreaks may lead to additional diseases such as meningoencephalitis in India and fatal encephalitis in Italy and La Reunion. The long-term effects include persistent joint pain and swelling. CHIKV outbreaks lead to increased costs in medical consultation (47%), hospitalization (32%), and drug consumption (19%) (Rezza and Weaver, 2019). These outbreaks result in significant economic losses, particularly in tourist destinations. It is crucial to develop a vaccine for CHIKV. This vaccine is vital for preventing epidemics and endemic conditions in various regions. Effective vaccination programs also reduce the number of cases and hospital admissions. Vaccination boosts economic growth in tourist destinations by attracting more visitors, facilitating travel, and enhancing developing nations’ health infrastructure and overall economy (Montalvo Zurbia-Flores et al., 2023). In clinical trials of IXCHQ, participants developed a protective antibody against CHIKV by day 28 after receiving a single dose of VLA1553. This finding suggested that a single intramuscular injection of VLA1553 is effective in preventing CHIKV infection (Valneva, 2023).

The World Health Organization (WHO) has two procedures for vaccine approval, which depend on the quality, safety, efficacy, and performance of the vaccine. These procedures include the WHO Emergency Use Listing (EUL) and WHO Prequalification (PQ). In the U.S., a vaccine can be approved for biologics licensure application (BLA) because of its safety, purity, and potency (US Food and Drug Administration, 2006). Additionally, there is a European equivalent called compassionate use. The WHO prioritizes vaccines listed in the PQ, coordinating with UNICEF and PAHO revolving funds and considering market demand. The vaccines are supplied to different countries following the recommendations of the WHO’s Strategic Advisory Group of Experts on Immunization (SAGE). In December 2021, a meeting of National Regulatory Agencies (NRAs) was convened by PAHO, ANVISA, and CEPI to discuss CHIKV vaccine developments and share regulatory experiences from the US FDA and EMA (US Food and Drug Administration, 2006; Cherian et al., 2023).

## 8 Future directions and challenges

The rapid spread of CHIKV and gaps in understanding its replication and the cause of arthritis persist, and the seasonal occurrence of Dengue and CHIKV suggests a potential need for further studies on the relationships of these diseases with climate (Caputo et al., 2020). Addressing the lack of specific CHIKV treatments involves developing alternative immunotherapies such as anti-CHIKV vaccines and monoclonal antibodies. Research gaps include exploring additional functions of IgG antibodies in natural CHIKV infections and assessing protective antibody responses in different populations (Simon et al., 2023). Protective monoclonal antibodies could serve as prophylactics, and their potential use with antiviral drugs is expected to increase. Understanding humoral immunity’s potential and dissecting ADE mechanisms are crucial for designing effective anti-CHIKV vaccines and ensuring the safety of therapeutic interventions in clinical trials (Chandley et al., 2023). The USFDA-approved single-dose, live attenuated CHIKV IXCHQ vaccine represents a significant step forward in preventing pandemics and endemics, but its efficacy has been proven for individuals aged 18 and above, and potentially fatal neonatal reactions from clinical trials remain uncertain (Idse et al., 2023).

## 9 Conclusion

Chikungunya remains a relevant public health problem, particularly in tropical areas of Africa, Asia, and Latin America but also in Europe (Alvarez et al., 2017), which has also faced autochthonous transmission during the last two decades due to travel and migration (Villamil-Gómez et al., 2015; Paniz-Mondolfi et al., 2016). Its prevention is crucial not only for acute clinical disease in adults and children (Villamil-Gómez et al., 2015; Rodriguez-Morales et al., 2016) but also because of the significant proportion of patients who develop its associated chronic disease (Alfaro-Toloza et al., 2015). A vaccine for such prevention is highly relevant. In this review, we highlighted the ongoing challenges posed by the rapid spread of CHIKV and emphasized the persistent gaps in understanding its replication and the cause of arthritis. The seasonal occurrence of Dengue and CHIKV underscores the potential importance of climate-related studies. Addressing the lack of specific CHIKV treatments requires the development of alternative immunotherapies, such as vaccines and monoclonal antibodies. Protective monoclonal antibodies hold promise as prophylactics, and their potential for combination with antiviral drugs is expected to increase. The recent approval of the CHIKV IXCHQ vaccine by the US FDA represents a significant step in prevention. However, challenges regarding efficacy in specific age groups and potential neonatal reactions underscore the need for continued research and vigilance in pursuing effective solutions.

## Author contributions

MS: Conceptualization, Methodology, Supervision, Visualization, Writing – original draft, Writing – review & editing, Investigation, Project administration, Software. MF: Conceptualization, Formal analysis, Funding acquisition, Methodology, Project administration, Software, Supervision, Visualization, Writing – original draft, Writing – review & editing. MK: Conceptualization, Data curation, Formal analysis, Investigation, Project administration, Resources, Supervision, Validation, Visualization, Writing – original draft, Writing – review & editing. SP: Conceptualization, Formal analysis, Funding acquisition, Project administration, Resources, Software, Supervision, Visualization, Writing – original draft, Writing – review & editing. IS: Conceptualization, Formal analysis, Investigation, Project administration, Resources, Software, Validation, Visualization, Writing – original draft, Writing – review & editing. AG: Conceptualization, Formal analysis, Funding acquisition, Methodology, Project administration, Resources, Supervision, Validation, Writing – original draft, Writing – review & editing. MA: Conceptualization, Formal analysis, Investigation, Methodology, Project administration, Resources, Supervision, Validation, Writing – original draft, Writing – review & editing. RS: Conceptualization, Data curation, Funding acquisition, Investigation, Project administration, Software, Supervision, Visualization, Writing – original draft, Writing – review & editing. RM: Data curation, Formal analysis, Investigation, Project administration, Software, Validation, Writing – original draft, Writing – review & editing. SS: Data curation, Formal analysis, Investigation, Methodology, Software, Visualization, Writing – original draft, Writing – review & editing. DB-A: Conceptualization, Formal analysis, Funding acquisition, Methodology, Resources, Supervision, Visualization, Writing – original draft, Writing – review & editing. CL: Investigation, Software, Supervision, Writing – original draft, Writing – review & editing. AR-M: Data curation, Funding acquisition, Investigation, Methodology, Resources, Software, Validation, Writing – original draft, Writing – review & editing.
